# Nuclear scaffold protein p54^nrb^/NONO facilitates the hypoxia-enhanced progression of hepatocellular carcinoma

**DOI:** 10.1038/s41388-021-01848-9

**Published:** 2021-06-02

**Authors:** Mengqin Shen, Ruixue Zhang, Wenzhi Jia, Zongping Zhu, Xiaoping Zhao, Li Zhao, Gang Huang, Jianjun Liu

**Affiliations:** 1 Department of Nuclear Medicine, Institute of Clinical Nuclear Medicine, Renji Hospital, School of Medicine, Shanghai Jiao Tong University, Shanghai, 200127 China; 2 Department of Nuclear Medicine, Qingdao Municipal Hospital (Group), Qingdao, 266011 China; 3 Shanghai Key Laboratory of Molecular Imaging, Shanghai University of Medicine and Health Sciences, Shanghai, 201318 China; 4 State Key Laboratory of Oncogenes and Related Genes, Shanghai Cancer Institute, Shanghai, 200127 China

**Keywords:** Cancer metabolism, Liver cancer, Tumour angiogenesis

## Abstract

Hypoxia and related oxidative stress are closely related to the development and treatment of hepatocellular carcinoma (HCC). However, the mechanism mediated by hypoxia in HCC has not yet been elucidated. Here, we found multifunction scaffold protein p54^nrb^/NONO exerted pleiotropic effects to regulate hypoxia transcription signals, thereby enhancing the progression of liver cancer. Extensive analysis of clinical data demonstrated that NONO was significantly upregulated and represented as a poor prognostic indicator of HCC. The crucial role of NONO in driving angiogenesis and glycolysis, two well-known cancer phenotypes mediated by hypoxia, was examined in vitro an in vivo. Mechanistically, NONO interacted with and stabilized both HIF-1 and HIF-2 complexes thus activating the transcription of hypoxia-induced genes. Besides, NONO bound pre-mRNA and subsequent mRNA of these genes to facilitate them splicing and mRNA stability, respectively. Thus, NONO knockout seriously disrupted the expression of a cluster of HIF-1/2 targets and impeded hypoxia-enhanced progression in HCC. In conclusion, NONO functioned as a multipurpose scaffold that interacted with HIF-1/2 complex and their downstream transcripts to facilitate the expression of hypoxia-induced genes, allowing malignant proliferation, indicating that NONO might be a potential therapeutic target for HCC.

## Introduction

Hepatocellular carcinoma (HCC) is the fourth most common cause of cancer-related death with increasing incidence worldwide [1]. The high mortality of liver cancers reflects the general ineffectiveness of current HCC therapies. It is generally believed that hypoxia plays important roles in hepatocarcinogenesis, HCC progression and re-occurrence after chemotherapy [2]. In fact, liver is originally vulnerable to hypoxia, which is attribute to the lower oxygen tension in the hepatic sinusoid while high metabolic rate of hepatocytes [3]. What’s more, secondary hypoxia resulted from common pathophysiological factors (e.g., viral infection, toxic substances exposure or inflammation) induces profound epigenetic/genetic alternations in hepatocytes [4]. These risk factors lead to repetitive-injury of hepatocytes and ultimately progress to HCC. The common therapeutic regimens of HCC include surgical resection, chemotherapy (e.g., sorafenib), interventional chemotherapy [e.g., transcatheter arterial chemoembolization (TACE)], and radiation. However, hypoxia or ischemia always accompanies and then follows these HCC therapies and heavily affects the therapeutic outcomes. In addition, hypoxic responses to therapy have important prognostic value for HCC [5, 6]. Therefore, deciphering the hypoxia-mediated mechanism of HCC will benefit a lot.

The best studied mechanism of response to hypoxia involves hypoxia inducible factors (HIFs). And HIF-1ɑ and HIF-2ɑ are well-characterized players in HCC hypoxic responses, which are stabilized and dimerized with HIF-1β to activate a multitude of genes. These target genes are involved in multiple aspects of tumorigenesis, including glucose metabolism, proliferation, cancer stem-like properties, angiogenesis, invasion, and metastasis [7]. Consequently, the activation of HIF-1/2 pathways are associated with aggressive tumor phenotypes and poor clinical prognosis. It is rational to add HIF-1/2 inhibitors to the cancer treatment regimens but the design of specific HIF-1/2 inhibitor is very challenging due to the complex upstream regulation and intertwined mechanisms. During the last decades, lots of HIF-1/2 inhibitors have been identified, but no selective HIF-1/2 inhibitor has been clinically approved, and parallelly, some approved drugs have demonstrated indirect inhibition for HIF-1/2 [8, 9]. Therefore, further research is needed to unravel the extensive complexity of HIF-1/2 regulation and develop a more precise anti-cancer treatment.

Although the transcriptional mechanism of HIF-1ɑ/β and HIF-2ɑ/β in activating hypoxia-inducible genes is prominent, other mechanisms are necessary to be coordinated that ensure swift and robust adjustment of protein expression levels in response to hypoxia. To regulate and integrate these multiple components and pathways throughout gene regulation, the cell needs factors that can bridging DNA, RNA, and protein. One such example of bridging proteins is the “multifunctional” Drosophila behavior/human splicing (DBHS) family. DBHS proteins possess both protein–protein and protein–nucleic acid binding sites that enable them to behave as a “molecular scaffold”. And DBHS proteins are frequently identified engaging in almost every step of gene regulation, including but not limited to transcriptional regulation and RNA processing [10]. A total of 54 kDa nuclear RNA- and DNA-binding protein/Non-POU-domain-containing octamer binding protein (p54^nrb^/NONO), one member of DBHS family, dysregulated in many types of cancers [11]. Our previous work demonstrated that NONO was upregulated in breast cancer and stimulated lipogenesis by interacting with and stabilizing sterol regulatory element-binding protein 1 (SREBP1) [12]. Soon afterwards Benegiamo et al. identified that NONO played a pivotal role in regulating the rhythmicity of genes involved in nutrient metabolism and maintaining the cellular energy homeostasis in the liver [13]. However, little is known about the functional role and the molecular mechanism of NONO in hypoxia-associated HCC progression.

In our study, we found NONO functioned as a multipurpose scaffold that tethered HIF-1/2 complex and transcripts of their targets to facilitate the transcription in response to hypoxia stimulus. We also found that NONO upregulated in HCC and was an adverse prognostic predictor for HCC. Given its significance in the HIF-1/2 signaling pathway, NONO might be a potential therapeutic target for HCC.

## Results

### NONO is a prognostic biomarker for HCC

**Fig. 1:**
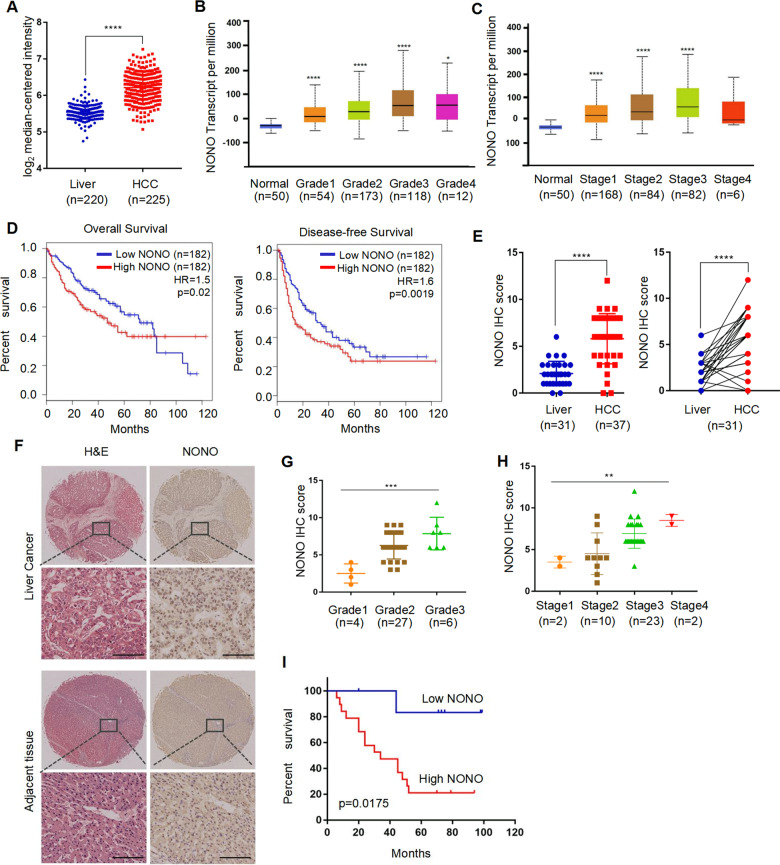
NONO is upregulated in HCC tissues and predicts poor prognosis. **A** Comparison of NONO mRNA expression between normal liver tissue (*n* = 220) and HCC (*n* = 225) from Oncomine dataset. **B**, **C** The mRNA level of NONO in different tumor grades (**B**) and TNM stages (**C**) of HCC. **D** Kaplan–Meier analysis showed elevated NONO mRNA was associated with both poor overall survival (OS) and disease-free survival (DFS) in HCC from GEPIA portal. **E** The expression of NONO protein between normal liver tissue and HCC (left) or in 31 paired HCC tissues (right) was analyzed by IHC. **F** Representative HE (left) and IHC (right) images of NONO expression in HCC and paired adjacent normal liver tissue. Scale = 100 μM (×200). **G**, **H** The protein level of NONO in different tumor grades (**G**) and TNM stages (**H**) of HCC. **I** The effect of NONO protein on 5-year survival rate of HCC patients was analyzed by Kaplan–Meier analysis.

To assess the clinical significance of NONO in cancer progression, we queried the Oncomine database and found NONO widely upregulated in different types of cancer (normal versus cancer), including hepatocellular carcinoma (HCC) (Figs. 1A and S1A). The RNA sequencing data of The Cancer Genome Altas (TCGA) from Gene Expression Profiling Interactive Analysis (GEPIA) platform also showed up-regulation of NONO positively correlated with poor overall survival rates in several types of cancer (Fig. S1B). In HCC patients with higher pathological grade and more advanced tumor stage, NONO mRNA levels were apparently increased (Fig. 1B, C). High-level of NONO in liver cancer was significantly related to poor overall survival or poor disease-free survival (Fig. 1D). In addition, we examined the relationship between NONO protein level and clinicopathological characteristics of 37 HCC sample. Consistent with previous NONO mRNA changes, we found the NONO protein level in HCC tissues was higher than the corresponding matched non-tumor tissues (Fig. 1E, F), and the NONO protein expression was also positively correlated with higher pathological grades or advanced tumor stages (Fig. 1G, H and Table 1). Survival analysis showed that patients with high NONO protein levels have a worse prognosis than patients with low NONO protein levels (Fig. 1I), representing as an independent prognostic indicator. These data indicate that NONO plays an important role in the progression of HCC.

**Table 1:** Analysis of correlation between NONO and clinic parameters of hepatocellular carcinoma.

Characteristics	All cases	Low	High	*P* value
Participants	37	10	27	
Age (years)				0.9023
<50	18	5	13	
≥50	19	5	14	
Gender				0.7975
Female	3	1	2	
Male	34	9	25	
HBV infection				0.0029
Negative	15	8	7	
Positive	22	2	20	
Hepatocirrhosis				0.7091
Negative	9	2	7	
Positive	28	8	20	
AFP (ng/mL)				0.8085
<20	16	4	12	
≥20	21	6	15	
Tumor size (cm)				0.0456
<5	16	7	9	
≥5	21	3	18	
^18^F-FDG SUV_max_				0.0095
<5.3	16	8	8	
≥5.3	21	2	19	
TNM				<0.0001
I	2	2	0	
II	10	7	3	
III	23	1	22	
IV	2	0	2	
Grade				0.0011
1	4	4	0	
2	26	6	20	
3	7	0	7	

### NONO exerts oncogenic activities in HCC in vitro and in vivo

**Fig. 2:**
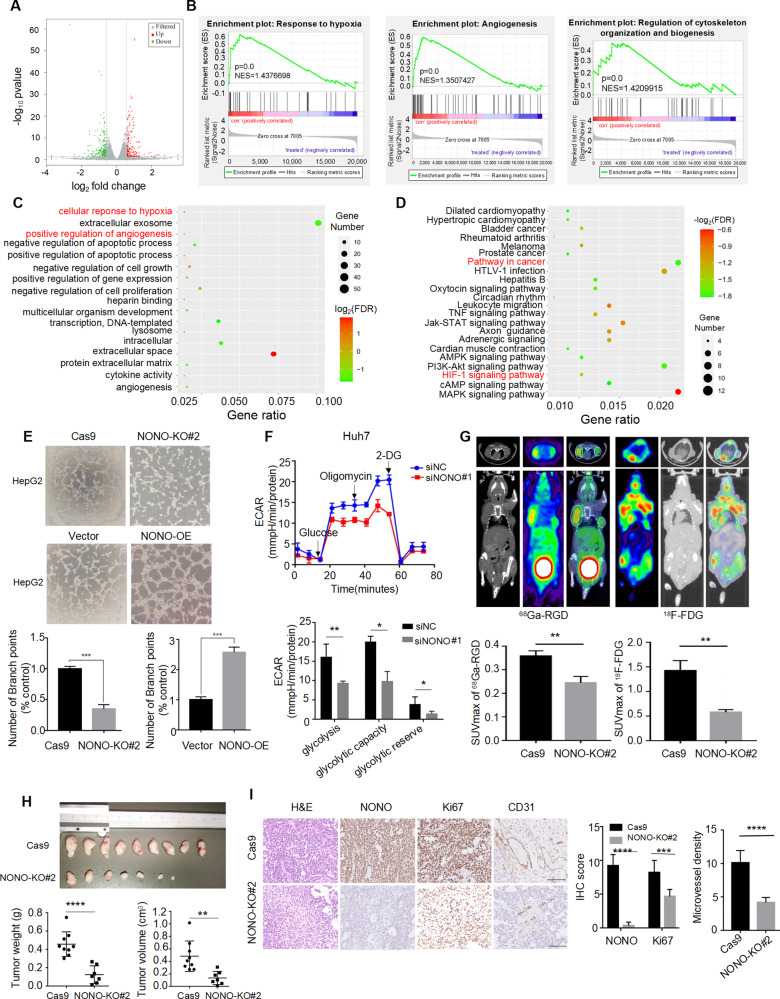
NONO exerts oncogenic activities in HCC in vitro and in vivo. **A** The volcano plot showed the differentially expressed genes (DEGs) (fold change ≥ 1.5, *p* < 0.05) in HepG2 cell with NONO knockdown by siNONO#1. **B**–**D** GSEA (**B**), GO (**C**) and KEGG pathway (**D**) analysis showed the differentially expressed genes (DEGs) were enriched in hypoxia response, angiogenesis, pathway in cancer, HIF-1 signaling pathway, etc. **E** Tube-formation assay was performed using HUVECs incubated with indicated condition mediums (CMs), less tube-formation was observed in NONO-KO groups (above) and more was found in NONO-OE groups (below). **F** The quantification of extracellular acidification rate (ECAR) was measured by Seahorse XF assays in Huh7 cells with NONO knockdown by siNONO#1. **G** ^68^Ga-RGD micro-PET/CT (left) and ^18^F-FDG (right) images of living nude mice were conducted 4 weeks after injection. Images showed obvious decrease uptake of ^68^Ga-RGD and ^18^F-FDG in NONO knockout group (left armpit) compared with the control group (right armpit). **H** The volume and weight of subcutaneous tumors were significantly diminished upon NONO knockout (*n* = 9). **I** The xenografts were subjected to H&E and IHC staining with anti-NONO, ki67 and CD31 antibodies. Scale bar = 100 μm (×200). The quantification of NONO and ki67 staining of subcutaneous tumors (*n* = 6) was analyzed. According to the staining status of CD31, microvascular densities were statistically analyzed (*n* = 6).

To clarify the oncogenic mechanisms of NONO in HCC, RNA sequencing (RNA-seq) was performed in HepG2 cells with NONO knockdown. The expression profiling data showed that there were 437 differentially expressed genes (fold change ≥ 1.5, *P* < 0.05, and FDR < 0.05), including 171 upregulated and 266 downregulated genes in total (Figs. 2A and S2A). GSEA analysis and GO enrichment analysis showed that NONO regulated the signal transduction networks related to angiogenesis and hypoxia response and cell membrane organization (Fig. 2B, C). KEGG analysis for differently expressed mRNAs demonstrated that NONO was closely associated with multiple cancer pathways (Fig. 2D), including MAPK pathway, PI3K/Akt pathway, HIF-1 pathway and Jak/STAT pathway. Next, we validated the NONO-related cancer phenotype in HCC cells. In the absence of NONO expression (Fig. S2B), the tube-forming ability of HCC cells was reduced, and vice versa (Fig. 2E). In addition, we observed that silencing NONO (Fig. S2C) evidently reduced glycolytic capacity of HCC cells (Fig. 2F). Further, we used PET tracer [^68^Ga]-NODAGA-RGD and ^18^F-FDG PET tracers were used to evaluate angiogenesis and glycolysis in vivo, respectively. The subcutaneous tumors formed by NONO knockout cells showed decreased uptake of [^68^Ga]-NODAGA-RGD and ^18^F-FDG (Fig. 2G) with smaller tumor volumes and lower tumor weights (Fig. 2H), indicating impaired angiogenesis and glycolysis. Consistently, NONO expression was highly correlated with the maximum standard uptake value (SUV_max_) in HCC patients who received ^18^F-FDG PET/CT imaging preoperatively (Fig. S2D). IHC staining of tumor tissue indicated that NONO-silenced tumors had lower ki-67 and CD31 expression (Fig. 2I). Altogether, these results indicated that NONO promoted HCC tumorigenesis in vitro and in vivo.

### NONO activates HIF-1/2 transcriptional network

**Fig. 3:**
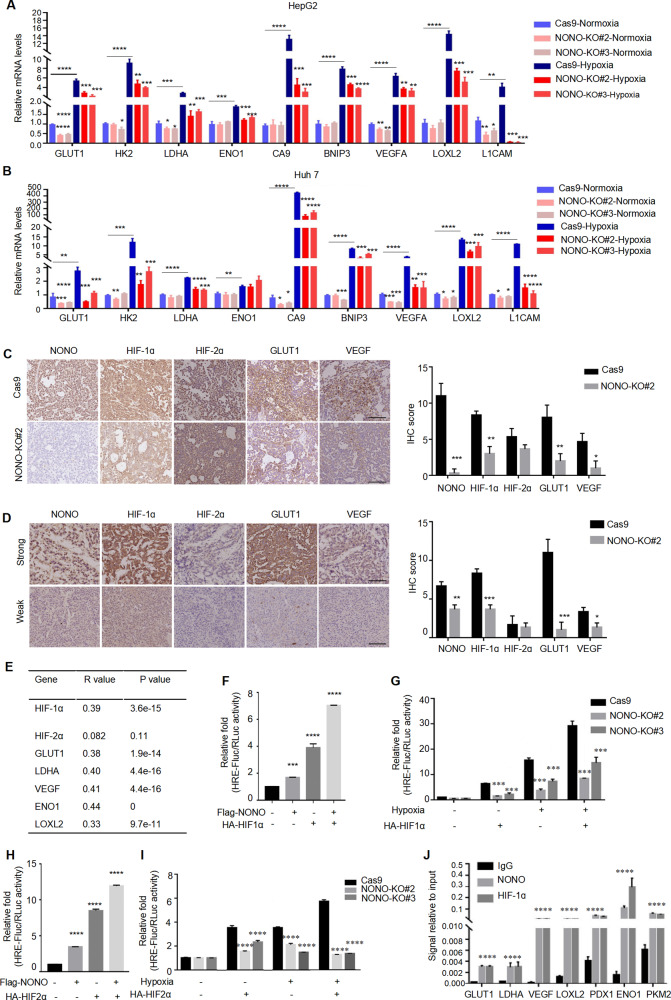
NONO is required to activate HIF-1/2 transcriptional network. **A**, **B** HepG2 cells (**A**) and Huh7 (**B**) with NONO knockout or not were treated with 1% O_2_ for 24 h. Then mRNAs were extracted and quantified by RT-qPCR with indicated primers. **C**, **D** The IHC staining of NONO and HIF-1α, HIF-2α, their shared targets (GLUT1, VEGF) were showed in the representative IHC images of subcutaneous tumor tissue (**C**) and HCC samples (**D**). **E** The Spearman correlation co-efficient were derived from GEPIA platform to show the correlation between NONO and HIF-1α, HIF-2α, GLUT1, LDHA, VEGF, ENO1, LOXL2. **F–I** Luciferase reporter plasmid inserted with four hypoxia-response elements (4 × HREs) was used in dual luciferase reporter assay. The dual luciferase reporter assays in HepG2 with NONO-overexpressing (**F**, **H**) or NONO-knockout (**G**, **I**) were presented as relative value with normalization against Renilla-Luc activity. **F**, **H** The luciferase reporter plasmid and Renilla luciferase plasmid were co-transfected with empty vector, NONO and HIF-1α (**F**) or HIF-2α (**H**) into HepG2 cells for the reporter assay. **G**, **I** Similarly, reporter assays were performed in NONO-knockout HepG2 cells transfected with HIF-1α (**G**) or HIF-2α (**I**) plasmids for 24 h, followed by hypoxia (1% O_2_) or normoxia treatment for 24 h. **J** Lysate from HepG2 cells with hypoxia treatment (1% O_2_, 24 h) were subjected to CUT&RUN assay. CUT&RUN assay products were quantified by qPCR with the indicated pairs of primers.

Based on NONO’s involvement in the regulation of hypoxia signaling pathways, we further analyzed the relationship between NONO and hypoxia responsiveness. There were four putative HIF-1 binding sites in NONO promoter region (Fig. S3A). However, the expression of NONO did not significantly change by neither hypoxia stimulus (Fig. S3B, C) nor HIF-1α/2α knockdown (Fig. S3D), indicating the HIF-1 binding sites in the NONO promoter appeared to be non-functional. Importantly, the levels of hypoxia-inducible genes including angiogenesis (VEGFA), metabolism reprogramming (GLUT1, LDHA, ENO1, CA9), mitochondrial function (BNIP3), invasion (L1CAM) and metastasis (LOXL2) were reduced upon NONO knockout (Fig. 3A, B). Immunohistochemistry analysis of mouse subcutaneous tumor (Fig. 3C) and HCC samples (Fig. 3D) showed low NONO expression had lower HIF-1α, HIF-2α, GLUT1 and VEGF expression, indicated that NONO was closely related to the HIF transcription network. What’s more, HIF-1ɑ (*r* = 0.39, *p* = 3.6e-15) and HIFs target genes VEGF (*r* = 0.41, *p* = 4.4e-16), GLUT1 (*r* = 0.38, p = 1.9e-14), LDHA (*r* = 0.4, *p* = 4.4e-16), ENO1 (*r* = 0.44, *p* = 0), and LOXL2 (*r* = 0.33, *p* = 9.7e-11) showed a positive correlation with the expression of NONO in the TCGA HCC provisional cohort (Fig. 3E), further confirming our findings. Ectopic expression of HIF-1α (3.90-fold) or NONO (1.68-fold) alone increased the transcription activity of HIF-1, while the co-expression of HIF-1α and NONO synergistically enhanced promoter activity (7.02-fold; Fig. 3F). And NONO knockout resulted in a significant reduction in HIF-1 transcriptional activity (Fig. 3G). Similar results were obtained from the effect of NONO on the transcriptional activity of HIF-2ɑ (Fig. 3H, I). We further analyzed whether NONO was directly involved in the HIF-mediated gene transcription process through CUT&RUN. The results showed that hypoxia-induced recruitment of NONO to promoter of a subset of the HIF-1/2 target genes (Fig. 3J). In summary, these data indicate that NONO enhances the transcriptional activity of HIF-1/2 and serves as a key regulator of the HIF transcription network.

### Hypoxia strengthens the interaction between NONO and HIF-1/2

**Fig. 4:**
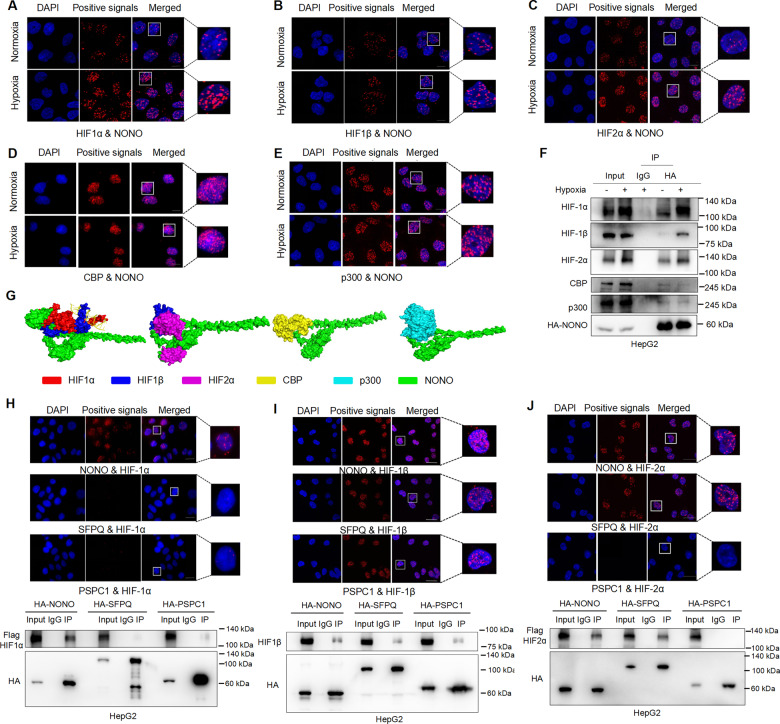
The interaction between NONO and HIF-1/2 was enhanced under hypoxia. **A–E** In situ proximity ligation assay (PLA) on Hep3B cells demonstrated the interaction between NONO and HIF-1ɑ (**A**), HIF-1β (**B**), HIF-2ɑ (**C**), CBP (**D**), and p300 (**E**) under normoxia or hypoxia. Positive PLA signals showed HIF-1ɑ/NONO complex (**A**), HIF-1β/NONO complex (**B**), HIF-2ɑ/NONO complex (**C**), CBP/NONO complex (**D**), or p300/NONO complex (**E**) which were shown as red clusters, and cell nuclei were counterstained with blue. Scale bar = 10 μm (×63). **F** HepG2 cells were transfected with HA-tagged NONO plasmid for 24 h, followed by hypoxia (1% O_2_) or normoxia treatment for 24 h. Finally, cells were collected to immunoprecipitated with anti-HA antibody, loaded for western blotting with anti-HIF-1α, HIF-2ɑ, HIF-1β, and CBP/p300 antibodies. IgG as negative control. **G** Computational docking models for human NONO (green) and HIF-1ɑ (red), HIF-1β (blue), HIF-2ɑ (purple), CBP (yellow), p300 (cyan) were predicted using ZDOCK. **H**, **J** The interaction between DBHS proteins and HIF-1ɑ (**H**) or HIF-1β (**I**) or HIF-2ɑ (**J**) in HepG2 cells was also determined by performing PLA (upper panel) and Co-IP (lower panel). For PLA assays, protein complexes were presented as red clusters, and cell nuclei were showed as blue ovals. Scale bar = 10 μm (×63). For Co-IP assays, cells were co-transfected with HA-tagged DBHS plasmids and Flag-tagged HIF-1ɑ (H) or HIF-2ɑ (J) for 48 h. Then cells were collected, immunoprecipitated with anti-HA antibody, loaded for western blotting with indicated antibodies. IgG as negative control.

Since NONO function as multifunctional scaffold protein by interacting with other proteins (including transcription factors), we hypothesized whether NONO regulates HIF-1/2 transcription activity through direct interaction with HIF-1/2. A series of protein–protein interaction assays based on co-immunoprecipitation and proximal ligand analysis showed that NONO has a robust interaction with HIF-1ɑ, HIF-2ɑ, HIF-1β and their central integrated co-activator CBP/p300 (Fig. 4A–F). It suggested that NONO activated the HIF-1/2 transcription network by interacting with HIF-1 complex and HIF-2 complex. In addition, hypoxia can significantly promote the interaction between NONO and HIF-1ɑ/β, HIF-2ɑ/β, but CBP/p300 expression did not show a similar effect (Fig. 4A–F). To evaluate the docking conformation of above protein complex, we used ZDOCK web server to perform protein–protein molecular docking. The ZDOCK scores for docking model were 1748.6, 1642.2, 1687.9, 1744.2 for HIF1ɑ/1β-NONO, HIF2ɑ/1β-NONO, CBP-NONO, p300-NONO respectively, suggesting a favorable and stable binding status (Fig. 4G). Considering DBHS protein rarely function alone as showed from structural and biological data, we wondered whether the other of DBHS members also involved in the HIFs complex and found SFPQ and PSPC1 slightly associated with HIF-1ɑ (Fig. 4H), obviously interacted with HIF-1β (Fig. 4I) and significant interaction between SFPQ and HIF-2ɑ (Fig. 4J). Collectively, NONO and also other DBHS members interact with both HIF-1 complex and HIF-2 complex.

### NONO stabilizes HIF-1 complex and HIF-2 complexes

**Fig. 5:**
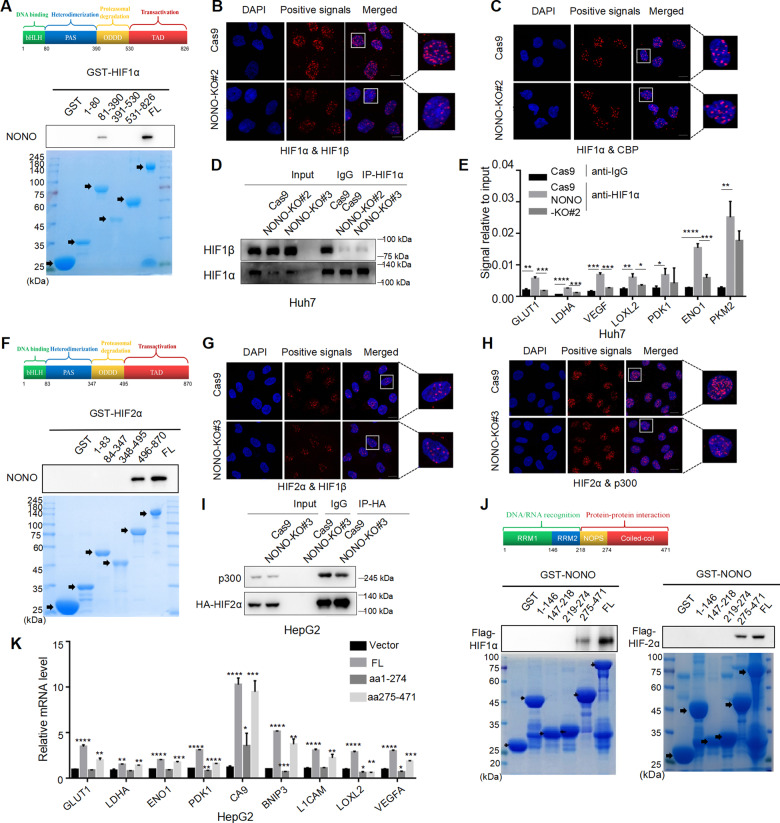
NONO stabilizes HIF-1 complex and HIF-2 complex. **A** HepG2 cell lysates were incubated with GST-fusion proteins of the indicated fragments of HIF-1ɑ in GST pull-down assays. **B**, **C** The effect of NONO on the stabilization of HIF-1ɑ/β complex (**B**) and HIF-1ɑ/CBP complex (**C**) in Hep3B was determined by performing PLA in the presence or absence of NONO. Protein complexes were presented as red clusters, and cell nuclei were showed as blue ovals. Scale bar = 10 μm (×63). **D** Huh7 cells with NONO knockout or not were treated with 1% O_2_ for 24 h, then was collected to be immunoprecipitated with anti-HIF-1ɑ antibody, finally loaded for western blotting with indicated antibodies. IgG as negative control. **E** Lysate from Huh7 cell with NONO knockout or not treated with 1% O_2_ treatment for 24 h was subject to CUT&RUN assay. CUT&RUN assay products were quantified by qPCR with the indicated pairs of primers. **F** HepG2 cell lysates were incubated with GST-fusion proteins of the indicated fragments of HIF-2ɑ in GST pull-down assays. **G**, **H** The effect of NONO on the stabilization of HIF-2ɑ/β complex (**G**) and HIF-2ɑ/p300 complex (**H**) in SMMC-7721 was determined by performed PLA in the presence or absence of NONO. Protein complexes were presented as red clusters, and cell nuclei were showed as blue ovals. Scale bar = 10 μm (×63). **I** HepG2 cells were transfected with HA-tagged HIF-2ɑ for 48 h, then cell lysates were collected to incubate with HA-tagged beads. The presence of HA-tagged HIF-2ɑ or endogenous p300 proteins were analyzed by immunoblotting. IgG as negative control. **J** HepG2 cell was transfected with Flag-tagged HIF-1ɑ (left) or HIF-2ɑ (right) plasmids for 48 h, then cells were collected and incubated with indicated fragments of NONO in GST pull-down assays.> **K** HepG2 cells with NONO-knockout were transfected with empty vector, full-length of NONO or truncated mutant (aa1-274 or aa275-471) for 24 h, followed by hypoxia treatment (1% O_2_) for 24 h. Finally, the relative expression of HIF-1/2 target genes were determined by RT-qPCR.

To further explore how these factors interacted functionally, we delineated the structural determinants for the association between NONO and HIF-1α, HIF-2α. First co-immunoprecipitation assays were performed with a series of HIF-1ɑ truncated fragments. We found that the PAS domain was necessary for their interaction (Fig. 5A). Since the PAS domain is responsible for functional dimerization, we then investigated the effect of NONO on HIF-1 complex formation. Knockout of NONO significantly reduced the interaction between HIF-1ɑ and HIF-1β but CBP/p300 was not (Fig. 5B–D), thus resulted in decreasing ability of HIF-1α to bind to the promoter sequence of its target genes (Fig. 5E). The same method was applied to map HIF-2α domain required for NONO and found the TAD of HIF-2α responsible for the interaction with NONO (Fig. 5F). Since TAD was critical function domain for HIF-2α transactivation, we speculated NONO may regulate the interaction of HIF-2α and CBP/p300. As expected, the interaction between HIF-2α and p300 was reduced in the absence of NONO but the stability of HIF-2ɑ/1β complex wasn’t affected (Fig. 5G–I). In addition, we found that both HIF-1α and HIF-2α were associated with the coiled-coil domain of NONO (Fig. 5J). To further explore functional relevance of the HIF-1α/NONO, HIF-2α/NONO interaction, the full-length and deletion mutant NONO (aa1-274) were overexpressed in NONO-KO cells. Compared with full-length of NONO, the ability of HIFs binding-deficient mutant NONO (aa1-274) to induce HIFs target genes was severely impaired (Fig. 5K). Taken together, NONO, as a co-activator of HIF-1/2, promotes the HIF-1/2 transcription network by regulating the dimerization of HIF-1 complex and the transactivation of HIF-2 complex.

### NONO facilitates the splicing mature and stabilizes hypoxia-induced genes

**Fig. 6:**
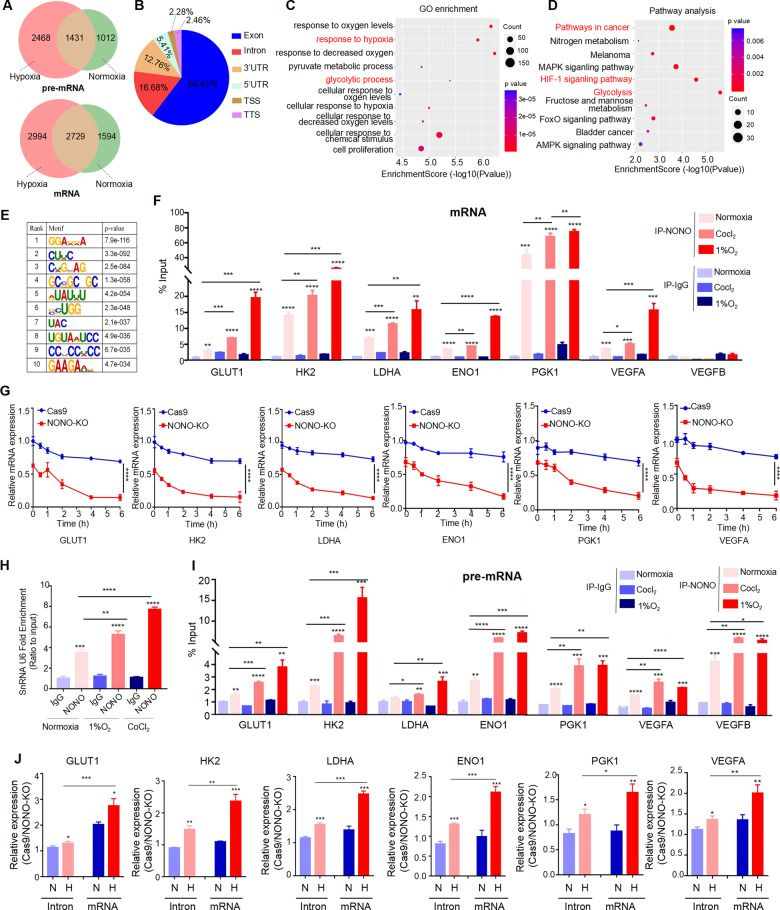
NONO facilitates the splicing mature and stabilizes hypoxia-induced genes. **A** Number of transcripts bound by NONO under normoxia and hypoxia in HepG2. **B** Distribution of NONO RIP-sequencing peak annotation for different regions. **C**, **D** Gene enrichment of biological process (**C**) and enriched KEGG pathways (**D**) analysis for the NONO-bound mRNA. **E** Significantly enriched RNA motifs among the RIP-seq. **F** Lysate from HepG2 cells after 1% O_2_ or Cocl_2_ (100 μM) treatment for 24 h was subjected to RIP assay, then RIP assay products were extracted by trizol. The mRNA bound by NONO were quantified by RT-qPCR with the indicated pairs of primers. **G** HepG2 cells under hypoxia were treated with actinomycin D (2 μM) and harvested at the indicated time points, then RNA was extracted from these cells and quantified by RT-qPCR with the indicated pairs of primers. **H**, **I** Lysate from HepG2 cells after 1% O_2_ or Cocl_2_ (100 μM) treatment for 24 h was subjected to RIP assay, then RIP assay products were extracted by trizol. The snRNA U6 (**H**) and pre-mRNA (**I**) bound by NONO were quantified by RT-qPCR with the indicated pairs of primers. **J** HepG2 cells with NONO knockout or not were treated with hypoxia (1% O_2_, 24 h) or normoxia. The expression ratio of Cas9 and NONO-KO group in intron and mRNA of indicated genes were determined by RT-qPCR.

Transcription and RNA processing are intimately interconnected [14]. As previously reported, transcriptional activation by NONO involves not only the interaction with transcription factor, but also the binding and processing of the nascent RNA transcript. Therefore, we performed RNA immunoprecipitation-sequencing (RIP-seq) to identify the NONO-associated transcriptomes in HepG2 cells under normoxia and hypoxia. The number of transcripts bound by NONO significantly increased upon hypoxic stimulation (Fig. 6A). Most identified RNAs were mature RNA (53.4%) and pre-mRNA (35.9%), with some noncoding RNAs (10.7%). And about 60.41% of NONO binding sites were within exons (Fig. 6B). GO enrichment analysis of biological processes and KEGG enrichment analysis demonstrated these mRNAs bound by NONO enhanced under hypoxia were enriched in HIF-1 pathway, response to hypoxia, glycolysis, etc. (Fig. 6C, D). In silico analyses showed that putative binding-site motifs were significantly GC-enrich (Fig. 6E). RIP quantitative RT-PCR were confirmed the binding of NONO to these mRNAs later, including GLUT1, HK2, LDHA, ENO1, PGK1, VEGFA (Figs. 6F and S4A). The reduced stability of these transcripts in NONO knockout cells was verified by RT-qPCR, using actinomycin D to inhibit transcription (Fig. 6G). Previous studies show NONO as spliceosome-associated protein and is important for pre-mRNA splicing. We found snRNA U6 bound by NONO was increased upon hypoxia stimulation, suggesting splicing activity of NONO may enhanced under hypoxia (Fig. 6H). What’s more, pre-mRNA of above transcripts bound by NONO were enhanced after hypoxia treatment (Fig. 6I). Next, we explored whether NONO involved in splicing mature of above transcripts. We measured their expression after hypoxia treatment in NONO-KO cell and its control. Compared with normoxia, the ratio of cas9 and NONO-KO group in intron was higher under hypoxia, suggesting that NONO regulated these genes transcriptionally upon hypoxia stimulus. Importantly, the ratio in mRNA was also increased under hypoxia and the extent of increase was higher than changes in intron, demonstrating that NONO regulated these genes post-transcriptionally in response to hypoxia stress (Fig. 6J). These results showed NONO promoted the expression of HIF targets by post-transcriptional regulation.

### Hypoxia-mediated tumor phenotypes can be overcome by NONO knockout

**Fig. 7:**
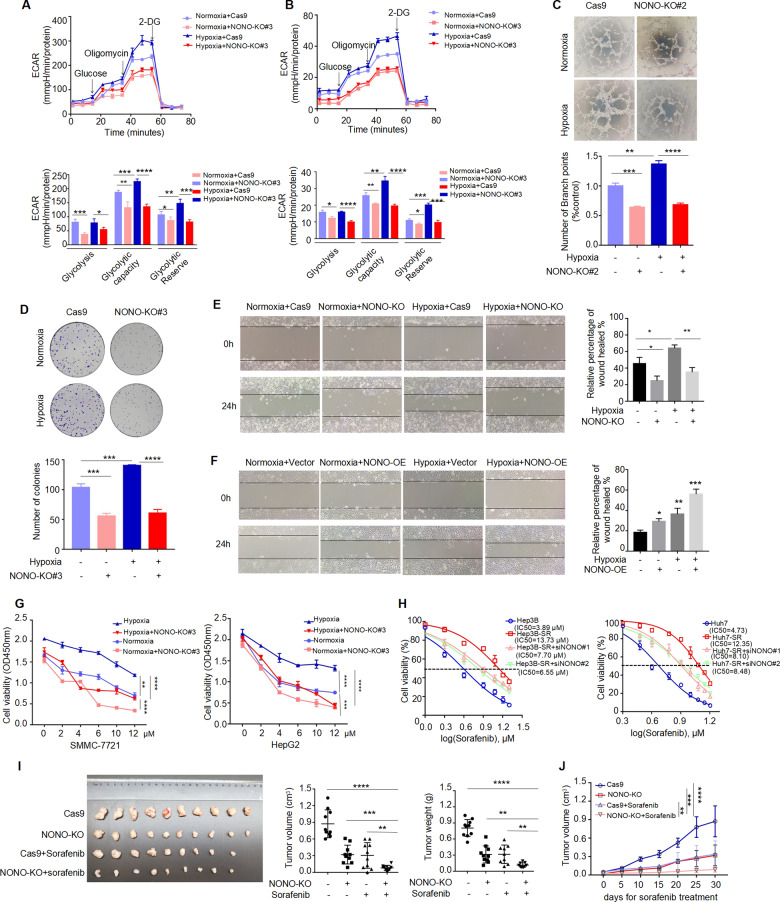
Hypoxia-mediated tumor phenotypes can be overcome by NONO knockout. **A**, **B** Extracellular acidification rate (ECAR) of HepG2 (**A**) and SMMC-7721 (**B**) cells with NONO knockout or not were determined by Seahorse XF assays after treating with Cocl_2_ (100 μM) for 24 h or not. **C** In vitro tube formation assays incubated with condition medium from indicated cells. The number of branch points quantified by image J. **D** The cell viability HepG2 cells with NONO knockout or not under hypoxia or normoxia was determined by colony formation assay. **E** The wound-healing assay in SMMC-7721 cell with NONO-KO or not was determined after hypoxia or normoxia treatment. The area of wound healed was measured by image J. **F** The wound-healing assay in Huh7 cell with NONO-overexpressing or not was determined after hypoxia or normoxia treatment. The area of wound healed was measured by image J. **G** HepG2 cells with NONO knockout or not were treated with sorafenib at indicated concentration for 48 h in the presence or absence of oxygen, then the cell viability was determined by CCK-8 assay. **H** Enhanced response to sorafenib due to NONO knockdown was observed in sorafenib-resistant Hep3B (left) and Huh7 (right). The IC_50_ of each treatment is presented. **I** The volume and weight of subcutaneous tumors in different treatment groups was determined (*n* = 10). **J** Mice bearing tumors xenografts were treated as described in “Materials and Methods”. Curves of tumor growth in each group were measured.

Since NONO played important role in cell hypoxia response, we detected whether NONO affected hypoxia-mediated tumor phenotypes. We observed glycolysis (Fig. 7A, B) and angiogenesis (Fig. 7C) ability were failed to enhance under hypoxia in NONO knockout cell. Consistently, the ability of colony formation enhanced by hypoxia was not significantly strengthened in NONO knockout cell (Fig. 7D). As we known, hypoxia governs the metastatic potential of multiple primary cancers [15]. We wondered if hypoxia-enhanced cell migration was dependent on the expression of NONO. Compared with control group, NONO knockout impeded hypoxia-induced cell migration (Fig. 7E) and NONO overexpression synergistically enhanced hypoxia-induced cell migration (Fig. 7F). In addition, sorafenib resistance was acquired partly attributed to hypoxic microenvironment [16]. We observed NONO knockout enhanced the growth inhibition effects of sorafenib, which were more pronounced under hypoxia compared with normoxia (Fig. 7G). To further understand the role of NONO in chemoresistance, sorafenib resistant (SR) HCC lines were established and CCK-8 assay was performed for drug resistance evaluation. As shown in Fig. 7H, resistant cells showed great improvement of sorafenib resistance after NONO knockdown. In addition, we observed that either single knockout of NONO or sorafenib treatment could inhibit tumor growth. More importantly, the combination of NONO knockout and sorafenib treatment resulted in the most significant inhibition of tumor growth (Fig. 7I, J), suggesting NONO knockout enhanced the therapeutic efficacy of sorafenib in vivo. Altogether, NONO knockout can reverse hypoxia-mediated malignant phenotypes of HCC.

## Discussion

**Fig. 8:**
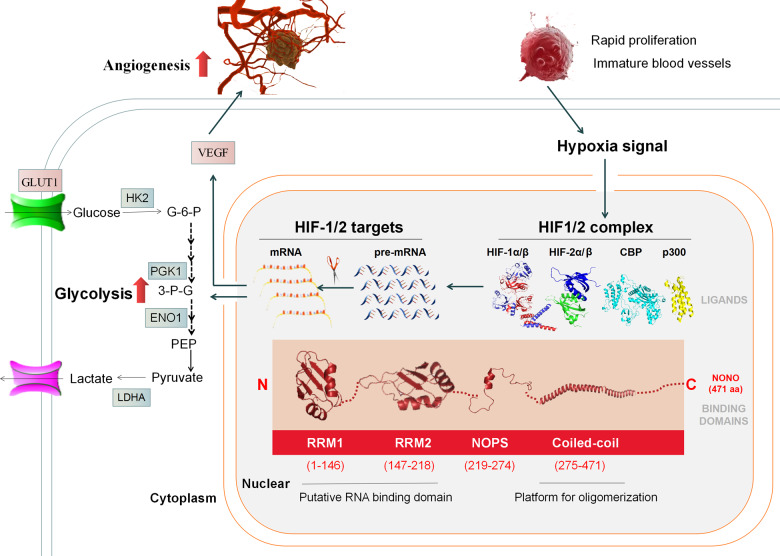
Schematic mechanism of NONO in hypoxia-enhanced progression of HCC. Schematic illustration of mechanisms for the expression regulation of hypoxia-induced genes by NONO thus enhancing angiogenesis and glycolysis.

The solid cancer progression is always accompanied with tumor hypoxia. Several advanced human cancers share HIF-1/2 activation as a final common pathway [7]. Hypoxic cells counter stress by referring to stimulus-varying regulatory network of gene expression. These molecular changes allow cells to adapt the stress by broadening the source of oxygen via angiogenesis and economizing oxygen consumption through a shift to glycolysis rather than oxidative metabolism. Here, we showed multipurpose molecular scaffold NONO transcriptionally and post-transcriptionally regulated the expression of hypoxia-induced genes by protein–protein and protein–nucleic acid interactions, respectively. The roles of NONO were determined by our in vitro and in vivo experiments that demonstrated it as the angiogenesis- and glycolysis-promoting effector (Fig. 8). Collectively, our results suggested NONO functioned as a potential prognostic and therapeutic marker of HCC and inhibition of NONO may help the disease management.

As molecular scaffold, DBHS proteins associate with multiple transcription factors and act bifunctionally as positive and negative transcriptional regulators. However, transcriptional activation by DBHS proteins appears to be driven by NONO. And transcriptional activation by NONO is always involved in a synergistic interaction with other activators [10]. We previously found NONO interacted with SREBP1a, the core transcription factor involved in lipid biogenesis, thus contributing to the lipogenesis of breast cancer [12]. Here, we showed that NONO interacted with HIF-1ɑ/2ɑ and associated with the promoters of HIF-1/2 targets, playing a critical role in the expression of hypoxia-induced genes. As we known, transcription of hypoxia-responsive genes is not solely dependent on HIF-1/2ɑ binding and other transcriptional co-factors are also required to achieve maximal transcription activation in response to hypoxia, such as NONO we found. This part was fully confirmed by the evidence that co-expressing NONO and HIF-1ɑ/HIF-2ɑ synergistically enhanced the HIF-1/2 reporter activity. DBHS members were reported to interact reciprocally and always be fundamentally dimeric in past literature [10], our study showed that all of them involved in HIF-1 while only NONO, SFPQ interacted with HIF-2. Actually, H Choudhry et al. reported hypoxia-induced paraspeckle in HIF-2ɑ-dependent manner was important for tumorigenesis [17]. Paraspeckle consists of 40 paraspeckle-associated proteins and structural backbone long noncoding RNA NEAT1, is a multifunction nuclear structure that sequesters transcriptionally active proteins and RNA transcripts to regulate gene expression. Both SFPQ and NONO are essential for the formation and integrity of paraspeckle [18, 19]. These data suggest that NONO may function as paraspeckles in the transcription regulation during hypoxia and further study needs to explore the function of paraspeckle in stimulus response. Mechanistically, we found the C-terminal end of NONO was responsible for its interaction with both HIF-1α and HIF-2α, which was showed as an extended α-helical “arm” projecting out from the main structure body in molecular docking model. The C-terminal end of NONO is characterized by high charge and provides an interface for dimerization and oligomerization [20]. Therefore, overexpression of truncation of this coiled-coil region failed to rescue HIF-1/2 targets expression in NONO knockout cells.

As for another important aspect of molecular scaffold, DBHS proteins exert typical function of RNA binding protein (RBP) based on their highly conserved tandem N-terminal RNA recognition motifs (RRMs), including transcript splicing, polyadenylation and stabilization, even location and translation. Mainly in the past two decades, RBPs have already earned the right to be in the spotlight, which attribute to their pivotal roles in post-transcriptional events and promising translation potential [21, 22]. While conventional anti-cancer therapies usually target one pathway at a time, targeting key RBP would affect more than one cancer hallmark and thus inhibiting cancer to the greatest extent. What’s more, the disruption RBP-RNA interactions by using small-molecule inhibitors or oligonucleotides have been already achieved, with favorable functional outcomes [23–25]. Meanwhile, synthesis of RBP that combines effectors domain with RNA binding domains (RBDs) to modulate a subset of cancer-associated transcripts is becoming a tangible reality [26]. Numerous original researches have showed NONO exerted oncogenic activity as a versatile RBP. Kim et al. found NONO bound and stabilized the mRNA of STAT3 thus contributing to the growth and chemotherapy resistance of triple-negative breast cancer (TNBC) [27]. Hu et al. demonstrated NONO promoted HCC progression via alternative splicing of bridging integrator 1 (BIN1) [28]. Here, we showed that NONO bound a cluster of HIF-1/2 targets under hypoxia and facilitated their splicing and enhanced subsequent mRNA stability. Like almost half of RBPs, NONO associates with RNA sites are also apparently “non-specific”, as putative binding-site motifs are different from previous studies. This “non-specificity” could be explained by various factors such as RBP/RNA concentration, RBP affinity distribution, rate constants for RNA substrate binding/dissociation, and RBP synergistic co-factors [29]. This binding feature enables conformational flexibility and adaptability to different structures but also brings disruption RBP-RNA interaction challenge. To sum up, the mechanism that NONO allows the integration the above-mentioned transcription events with these RNA processing machineries, which termed “Co-transcriptional RNA processing”, makes mRNA production temporally and spatially convenient [14]. This molecular mechanism may ensure cells efficiently and promptly produce the precise repertoire of stress-responsive factors.

Interestingly, we found four HIF-1 binding sites in NONO promoter but all appeared to be non-functional. Gregg L. Semenza et al. previously reported that hypoxia response elements (HREs) in ALDA and ENO1 promoters were functionally-essential HIF-1 binding sites, but other HIF-1 binding sites in the same genes made no contribution to the hypoxic response, indicating that presence of HIF-1 binding site was necessary but not sufficient for HRE function. Several mechanisms might involve in functional and non-functional HIF-1 sites: (i) Location of site; (ii) Relative binding affinity; (iii) Binding of constitutive factors; (iv) Bipartite structure of HREs [30]. Our results showed NONO also regulated biological phenotypes of HCC, interacted with HIFs complex or hypoxia-induced transcripts under normoxia. In addition to low oxygen, HIFs are also regulated by many other stimuli (e.g., cytokines, metabolic intermediates, and cell growth signals), which is commonly referred to as “pseudohypoxia” [31]. Therefore, the effect of NONO on biological phenotypes was more likely HIF-dependent rather than hypoxia-dependent.

With roles in almost every step of gene regulation, it is not surprising that abnormal expression of NONO results in malignant transformation of normal cells. To date, emerging evidences have demonstrated that NONO was frequently overexpressed in various cancer and is an independent prognostic factor for some cancers [11]. Although NONO is functionally characterized and has profound impact on tumorigenesis in a spectrum of cancer types, only few has been fully characterized. Here, we described how NONO functioned as an oncogene in HCC progression. However, Xie et al. showed NONO inhibited lymphatic metastasis of bladder cancer [32]. Interestingly, NONO upregulated in ER (+) while decreased in ER (−) breast tumor [33]. It seems that the precise function of NONO depends on cell-type, exogenous stimuli, interaction partners, its own modification status even the time of day. Future study needs to take these elements into consideration.

As mentioned earlier in this article, many scientists struggled to target HIF-1/2 pathways but gained little. Cancer cells can still survive by compensatory enhanced activation of other pathways. For decades, directly targeting the downstream targets of these pathways such as GLUT1 and LDHA have been long recognized as potential treatment [34, 35]. However, it is still a critical question that how to simultaneously target multiple oncogenes to overcome drug-resistance to some degree. Now targeting such multipurpose protein is expected to break through the bottleneck. Kim et al. revealed auranofin as NONO inhibitor in TNBC by drug screening [27]. Auranofin is a safe and effective oral medicine of rheumatoid arthritis over 30 years and is recently reported as a potent anti-cancer agent through increasing cellular oxidative stress and inducing apoptosis [36]. Sorafenib was the only effective first-line drug approved for HCC. Sorafenib improved median overall survival but about 30% patients showed inherent or acquired drug resistance [37]. Our data showed NONO knockout enhanced the therapeutic efficacy of sorafenib. Recently, Kessler et al. showed an induction of paraspeckles in sorafenib-resistant HCC and purposed paraspeckle-associated protein NONO, PSPC1, and RBM14 might be promising targets [38], which strengthened our view about the importance of NONO in sorafenib resistance of HCC.

It’s been almost 30 years from NONO first identified from Hela cells [39]. Now we have a sound framework to reliably investigate this remarkably adaptable and versatile protein. However, our understanding of the precise mechanistic detail of the NONO under specific biological process is still not adequate. Better comprehension of the definite mechanism of NONO during the tumor-specific physio-pathological processes will make it therapeutically invaluable.

In summary, our study has revealed NONO functions as a multifunctional scaffold that assembles HIF-1 and HIF-2 protein complexes to activate transcription and interacts with subsequent transcripts of HIF-1/2 targets to facilitate their splicing and stability, which endow cancer cells with powerful adaptability and viability in hypoxia microenvironment. Therefore, NONO might be a potential therapeutical target for HCC.

## Materials and methods

### Cell lines and cell culture

See Supplementary information for details.

### siRNA, plasmid and transfection

See Supplementary Information for details. The sequence of siRNA oligos were listed in Table S1. The vectors of plasmids were summarized in Table S2.

### Lentivirus packaging and screening knockout cells

See Supplementary Information for details.

### RNA sequencing

See Supplementary Information for details.

### RNA immunoprecipitation and RIP-seq

See Supplementary Information for details. PCR primers for RIP assays were listed in Table S3.

### mRNA stability assay

See Supplementary Information for details.

### CUT&RUN assay

See Supplementary Information for details. PCR primers for CUT&RUN assay were listed in Table S4.

### Proximal ligand assay

See Supplementary Information for details.

### GST pull-down assay

See Supplementary Information for details.

### Molecular docking

See Supplementary Information for details.

### Animal experimentation

See Supplementary Information for details.

### Patient samples

See Supplementary Information for details.

### Tube formation assay

See Supplementary Information for details.

Extracellular acidification rate (ECAR) and oxygen consumption rate (OCR) assays, cell counting kit-8 and colony formation, Wound-healing assay, Co-IP, Western blot, real-time quantitative PCR, Dual luciferase reporter assay, and immunohistochemistry were as described previously [40, 41]. All antibodies were listed in Table S5. The primers for RT-qPCR were summarized in Table S6.

### Statistical analyses

See Supplementary Information for details.

## Supplementary information

Supplementary data

## Data Availability

Raw sequencing and processed RNA-Seq data from our study are not publicly available due to other unfinished researches but are available from the corresponding author on reasonable request.
