# Supplementary material for: Nuclear scaffold protein p54nrb/NONO facilitates the hypoxia-enhanced progression of hepatocellular carcinoma
Source: Oncogene. 2021 Jun 2;40(24):4167–83. doi: 10.1038/s41388-021-01848-9 (PMC8211563; doi:10.1038/s41388-021-01848-9)
Supplement: Supplementary file 1 — Supplementary data [file 41388_2021_1848_MOESM1_ESM.pdf]

Supplementary information

I. Experimental Procedures

Cell lines and cell culture

The human liver cancer cell lines HepG2, Huh7, Hep3B, LM3, 97L, SMMC-7721 were obtained from ATCC and cultured in Dulbecco's modified Eagle's medium (DMEM) (Gibco, Grand Island, NY, USA). The human umbilical vein endothelial cell (HUVEC) was purchased from FuHeng Biology (Shanghai, China) and cultured in Endothelial Cell Medium (ECM). The sorafenib-resistant cells were established by treating the cells with sorafenib (MCE) at a concentration just below their respective IC_{50} at the beginning, followed by a 15% increase in the sorafenib concentration every week until the maximum tolerated doses had been reached. All medium was supplemented with 10% fetal bovine serum (Gibco), 100 μg/mL penicillin (Gibco), and 100 μg/mL streptomycin (Gibco). All cells were STR authenticated, mycoplasma-free and cultured in a 37°C incubator with 5% CO2 and a humidified atmosphere.

siRNA, plasmid and transfection

SiRNA and negative control was designed and synthesized by GenePharma (Shanghai, China). The plasmids used in the study were all confirmed by DNA sequencing. Cells were transfected with siRNA and plasmid by using lipofectamine 2000 Reagent (Invitrogen) and Neofect™ DNA transfection reagent (Neofect biotech, Beijing, China) according to the instructions respectively. Cells were collected to perform functional assays after transfection 48h. The sequence of siRNA oligos in this study were listed in Table S1. The vectors of plasmids were summarized in Table S2.

Lentivirus packaging and screening knockout cells

We generated NONO knockout cell lines by CRISPR/Cas9-mediated genome editing [1]. The

single guide RNA (sgRNA) targeted coding sequence (CDS) of NONO were obtained from the published literature. Different sgRNAs were constructed into the plenti-CRISPR v2 (a generous gift from Dr. Feng Zhang) and was transfected into HEK293T cells coupled with three-plasmid system (Genechem Biotech, China). Lentiviral particles were harvested and condensed after transfection 48h. The cells infected with the lentivirus were screened by puromycin. Single colonies were isolated by limited dilution and expansion and validated by immunoblotting. The clone 2 was obtained from pLentiCRISPRv2-NONO-gRNA#4 and the clone 3 was obtained from gRNA-NONO#40.

RNA sequencing

Sample of approximately 1×10^6 HepG2 cells was transfected with NONO-targeted siRNA#1 and siNC for 48 hours respectively, and total RNA was extracted using TRIzol Kit (Ambion,15596-026). RNA sequencing was carried out in collaboration with Shanghai Oebiotech. Differentially expressed genes (DEGs) were identified using the DESeq R package. P value <0.05 and foldchange ≥1.5 was set as the threshold for significantly differential expression. GO enrichment and KEGG pathway enrichment analysis of DEGs were separately performed by using DVID v6.8 and figured based on R package. GESA analysis was performed by using GSEA_4.0.3 soft.

RNA Immunoprecipitation and RIP-seq

RNA immunoprecipitation (RIP) assays were performed using the Magna RIP™ RNA-binding protein immunoprecipitation kit (Millipore, Massachusetts, USA). We used NONO antibody to perform RIP assays with HepG2 cells after hypoxia treatment for 24h. RIP sequencing was carried out in collaboration with Shanghai Cloudseq. The high-quality reads were aligned to the human

reference genome (UCSC hg38) with hisat2 software. Then, guided by the Ensembl gtf gene annotation file, cuffdiff software (part of cufflinks) was used to get the FPKM as the expression profiles of pre-mRNA and mRNA, and fold change and p-value were calculated based on FPKM, differentially expressed pre-mRNAs and mRNA were identified. GO and Pathway enrichment analysis were also performed based on the differentially expressed pre-mRNAs and mRNAs. PCR primers for RIP assays were listed Table S3.

mRNA stability assay

Cells at 80% confluence were treated with 2 μg/mL actinomycin D (MCE) and collected at the indicated time points. Total RNA was extracted and mRNA levels of GLUT1, HK2, LDHA, ENO1, PGK1 and VEGFA were determined by RT-qPCR.

CUT&RUN Assay

Cleavage Under Targets & Release Using Nuclease (CUT&RUN) assay was performed by using CUT&RUN Assay Kit (Cell Signaling Technology, USA). Cells were immobilized on Concanavalin after hypoxia treatment 24h, permeabilized with digitonin, incubated with NONO antibody and pAG-MNase fusion enzyme overnight. The addition of Ca^{2+} activates the pAG-MNase to cleave and liberate the desired chromatin fragment. These DNA fragments was purified by DNA Purification Buffers and Spin Columns (Cell Signaling Technology, USA). Finally, DNA was quantified by qPCR. PCR primers were listed Table S4.

Proximal ligand assay

Proximity Ligation Assay (PLA) was performed by using the Duolink® In situ Red Starter Kit Mouse/Rabbit (Sigma-Aldrich). Briefly, cells were grown on glass coverslips, fixed with 4% paraformaldehyde for 15min, permeabilized with 0.1% Triton X-100 for 10min. After blocking,

the cells were incubated with pre-diluted primary antibodies overnight at 4℃. The subsequent step was incubating the pre-diluted anti-rabbit plus and anti-mouse minus probes at 37℃ for 1h. Then, samples were incubated with 1× ligation buffer containing ligase for 30 min at 37℃, then incubated with 1× amplification solution containing the polymerase for 100 min at 37°C. Finally, coverslips were mounted on the slide with Duolink® In situ Mounting Medium with DAPI. After approximately 15 minutes, slides were analyzed by confocal microscopy (Leica TCS SP8) using a 63× objective.

GST pull-down assay

GST-fused NONO or HIF-1α or HIF-2α full-length or mutants were expressed in E. coli BL21 and purified using Glutathione-Sepharose 4B beads (GE healthcare, Uppsala, Sweden) according to the standard protocols. A few proteins were used to verify its expression by Coomassie blue staining. Cell lysates were mixed with purified fused GST or GST-NONO beads at 4℃ for 1h. The beads were then washed 10 times with IP buffer. The resulting beads were analyzed by Western blots using specific antibodies.

Molecular docking

Homology-based structural modeling of NONO was performed using SWISS-MODEL web server (http://swissmodel.expasy.org) [2-4]. Human SFPQ (PDB ID: 4WIJ) was selected as templates for NONO (the sequence similarities are 73.6%). Human SFPQ (PDB ID: 4WIJ), Human HIF-1α/1β-HRE complex (PDB ID: 4ZPR), HIF-2α/1β complex (PDB ID: 6D0C), p300 (PDB ID: 4BHW) and CBP (PDB ID: 4OUF) were also downloaded from PDB (http://www.rcsb.org/). Molecular docking analyses were performed using ZDDOCK web server (http://zdock.umassmed.edu/). Binding affinities for those complexes were evaluated by ZDOCK

2.3.2 scoring function. Graphical representations of the docking structures were constructed by PyMOL software (ver. 1.3; DeLanoScientific, San Carlos, CA, US).

Animal Experimentation

Four-week-old BALB/c-nu/nu and NCG male mice were used for animal studies. All experimental procedures using animals were in accordance with the guidelines provided by the Animal Ethics Committee of Renji Hospital of Shanghai Jiao Tong University School of Medicine. Mice were inoculated subcutaneously with 1×10^7 NONO knockout Huh7 cells within matrigel in the right flank, while control cells in the left flank to establish the HCC xenograft model. At approximately 4 weeks, we monitored subcutaneous tumor of nude mouse tumor xenografts by ^{18}F-FDG or ^{68}Ga-RGD micro-PET/CT as previously described [5, 6]. After that all mice were euthanized and tumor tissue were harvested to stained with HE, NONO, Ki67, CD31, HIF-1α, GLUT1, LDHA and VEGF antibody. Additionally, mice were subcutaneously injected with Huh7 cells with NONO-KO (right armpit) or not (left armpit). After 10 days, mice were randomly divided into two groups and treated with sorafenib (50 mg/kg) orally once daily. The tumor volume was measured every 5 days by using calipers and calculated by the formula: $Volume=1/2\times length\times width^2$. Mice were sacrificed after sorafenib treatment for 30 days.

Patient samples

A group of 31 paired and another 6 cases of paraffin-embedded HCC tissues were used for IHC and prognosis analysis. All HCCs were firstly diagnosed without therapy treatment. All samples were acquired from the surgical specimen archives of Renji Hospital, School of Medicine, Shanghai Jiaotong University. Ethical approval was obtained from the Ethic Committee of Renji Hospital, School of Medicine, Shanghai Jiaotong University. Written informed consent was

obtained from each patient and the study was conducted in accordance with the International Ethical Guidelines for Biomedical Research Involving Human Subjects (CIOMS).

Tube formation assay

HUVEC tube formation assay was performed. Briefly, 10 μL of pre-cooled Matrigel (Corning, USA) was transferred to each well of a μ-Slide (ibdi, Martin Reid, Germany) and polymerized for 60 min at 37°C. HUVECs (2×10^4) in 50 μL of conditioned medium were added to each well and incubated at 37°C and 5% CO2 for 3h. Capillary tube structure was photographed under a ×100 bright-field microscope and quantified by measuring the total length of completed tubes. Each condition was assessed in triplicate.

Statistical analyses

All data were represented as mean±s.d. of three or more independent experiments. Student's t test or one-way analysis of variance (ANOVA) followed by Dunnett's multiple comparisons test was performed to evaluate differences between two groups or more than two groups, respectively. Pearson's correlation was performed to analyze the correlation between genes mRNA and protein levels. Survival rates were determined using the Kaplan-Meier method (log-rank test). P value of <0.05 was considered statistically significant. All statistical analyses and figures were generated using GraphPad Prism 7 software. (*P < 0.05, **P < 0.01, ***P < 0.001, ****P < 0.001)

References

1. Lahaye X, Gentili M, Silvin A, Conrad C, Picard L, Jouve M, et al. NONO Detects the Nuclear HIV Capsid to Promote cGAS-Mediated Innate Immune Activation. Cell 2018;175:488-501.e22.

2. Waterhouse A, Bertoni M, Bienert S, Studer G, Tauriello G, Gumienny R, et al.

SWISS-MODEL: homology modelling of protein structures and complexes. Nucleic acids research 2018;46:W296-w303.

3. Bienert S, Waterhouse A, de Beer TA, Tauriello G, Studer G, Bordoli L, et al. The SWISS-MODEL Repository-new features and functionality. 2017;45:D313-d9.

4. Guex N, Peitsch MC, Schwede T. Automated comparative protein structure modeling with SWISS-MODEL and Swiss-PdbViewer: a historical perspective. Electrophoresis 2009;30 Suppl 1:S162-73.

5. Shen M, Zhao X, Zhao L, Shi L, An S, Huang G, et al. Met is involved in TIGAR-regulated metastasis of non-small-cell lung cancer. Molecular cancer 2018;17:88.

6. Hua Q, Jin M, Mi B, Xu F, Li T, Zhao L, et al. LINC01123, a c-Myc-activated long non-coding RNA, promotes proliferation and aerobic glycolysis of non-small cell lung cancer through miR-199a-5p/c-Myc axis. Journal of hematology & oncology 2019;12:91.

II. Supporting Figures

Figure S1

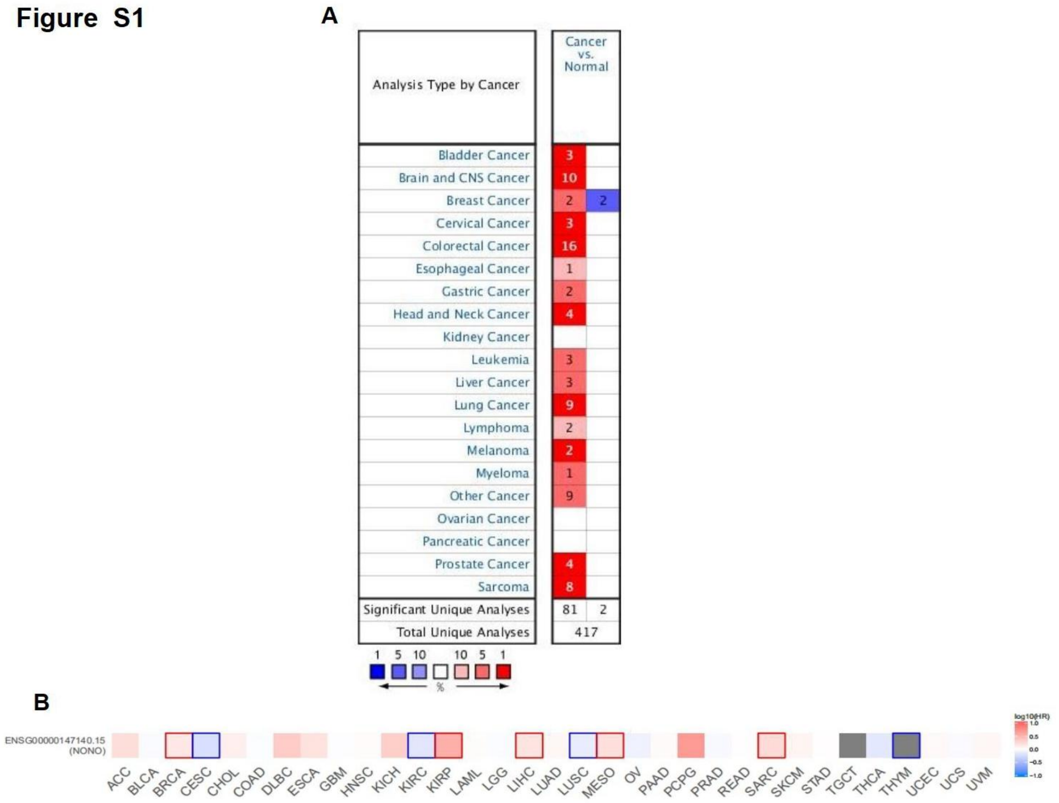

Supporting FIG.S1. NONO is upregulated and predicts poor cancer prognosis.

(A) The graph was a representation of the Oncomine dataset with significant mRNA overexpression (red) or reduced expression (blue) of NONO gene (cancer vs normal).

(B) The survival map showed upregulated expression of NONO have significant poor prognosis in BRCA, KIRP, LIHC, MESO and SARC, reverse results in CESC, KIRC and LUSC. BRCA, Breast invasive carcinoma. KIRP, kidney renal papillary cell carcinoma. LIHC, Liver hepatocellular carcinoma. MESO, Mesothelioma. SARC, Sarcoma. CESC, Cervical squamous cell carcinoma and endocervical adenocarcinoma. KIRC, Kidney renal clear cell carcinoma. LUSC, Lung squamous cell carcinoma.

Figure S2

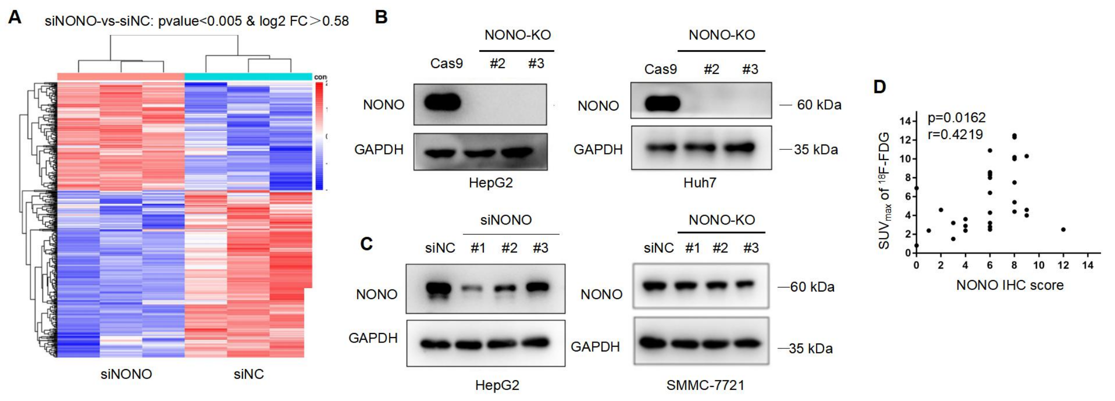

Supporting FIG.S2. NONO exerts oncogenic activities in HCC in vitro and in vivo.

(A) Hierarchical clustering analysis of differentially expressed genes in RNA-seq analysis.

(B) The NONO knockout efficiency by CRISPR-Cas9 was confirmed by Western Blot in HCC cells.

(C) The knockdown efficiency of NONO by siRNA was determined by Western Blot in HCC cells.

(D) The correlation between NONO expression and SUV_{max} of ^{18}F-FDG in HCC sample. SUV_{max}, maximum standard uptake value.

Figure S3

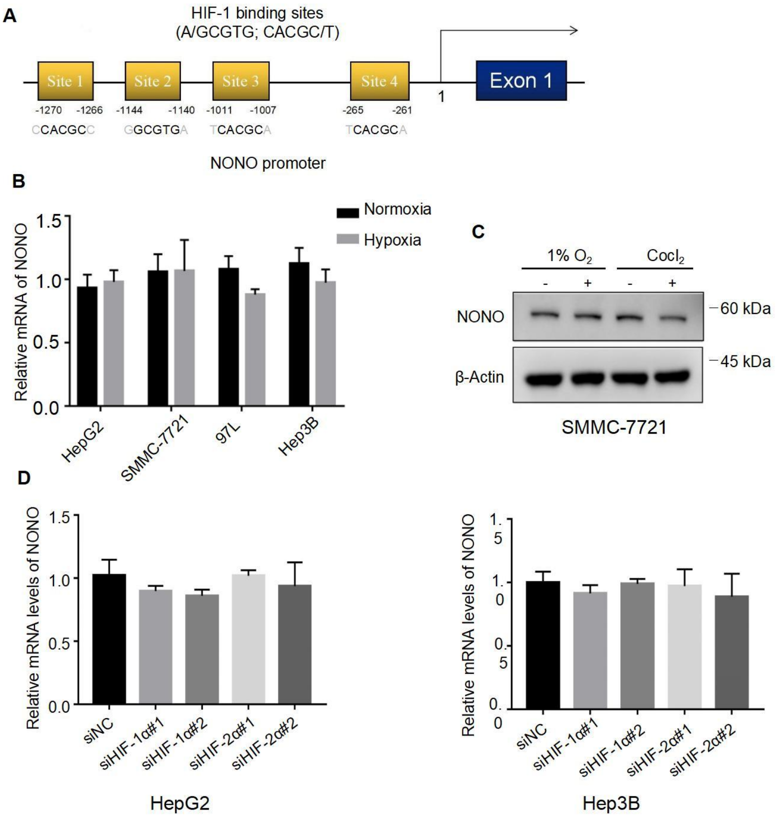

Supporting FIG.S3. The effect of hypoxia on the expression of NONO mRNA.

(A) The graph showed HIF-1 binding sites located in promoter region of NONO.

(B-C) The mRNA (B) and protein (C) of NONO were determined in various HCC cells treated with hypoxia (1% O_2) or normoxia for 24h.

(D) HepG2 (left) and Hep3B (right) cells were treated under hypoxia (1% O2) or normoxia for 24h after being transfected with siHIF-1α or siHIF-2α or negative control for 24h. Finally, the mRNA level of NONO was quantified by RT-qPCR.

Figure S4

A

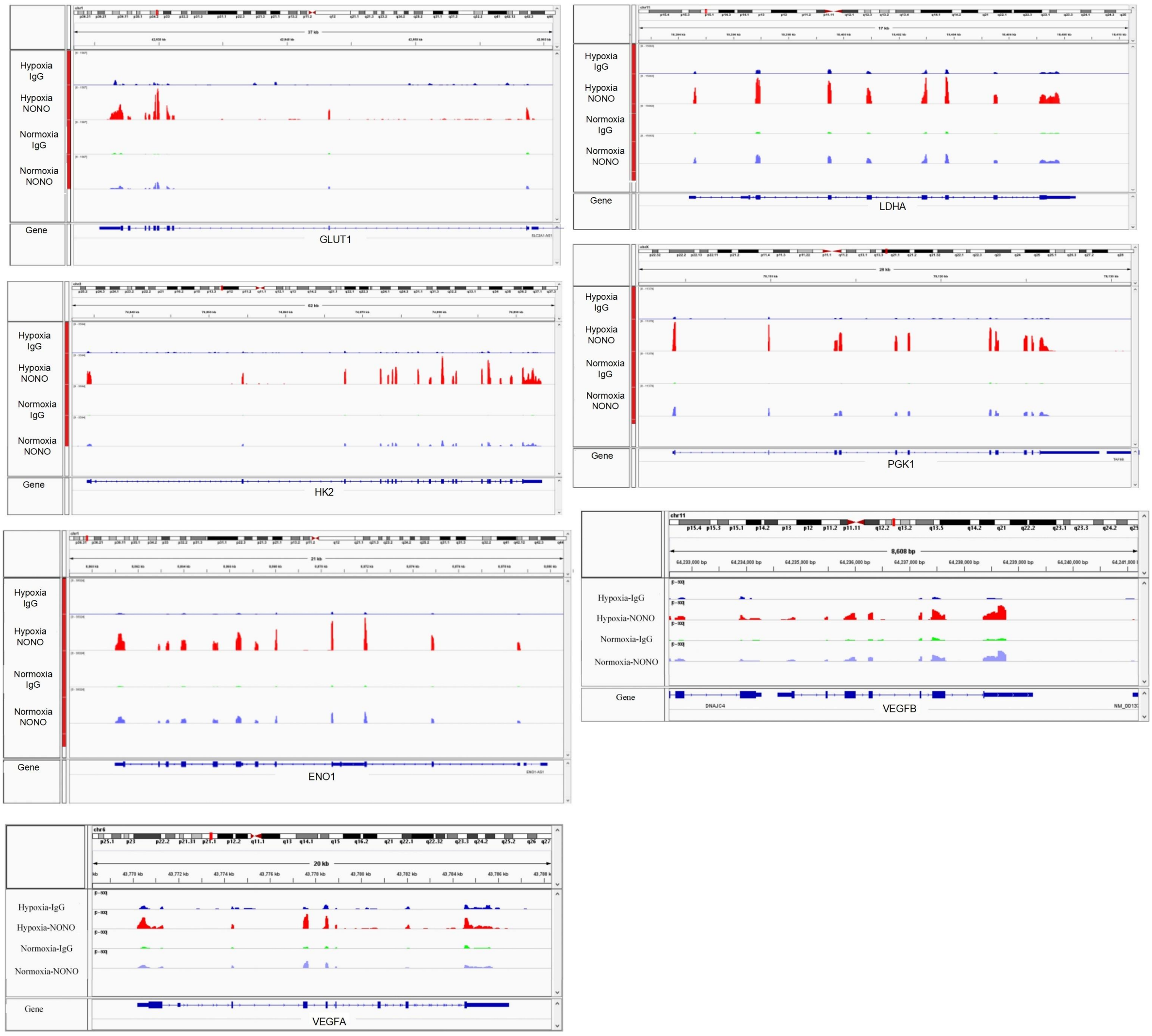

Supporting FIG.S4. The RIP-seq reads mapping to HIF1/2 targets.

(A) IGV software view of the RIP-seq reads mapping to GLUT1, HK2, LDHA, PGK1, ENO1, VEGFA and VEGFB gene.

III. Supporting Tables

Table S1. Sequences of siRNAs

Type	Sequence (5’-3’)
siNONO#1	CAGGCGAAGUCUUCAUUCAUA
	UAUGAAUGAAGACUUCGCCUG
siNONO#2	GCCAGAAUUCUACCCUGGAAA
	UUUCCAGGGUAGAAUUCUGGC
siNONO#3	UCCAGAGAAGCUGGUUAUAAA
	UUUAUAACCAGCUUCUCUGGA
siHIF-1α#1	GUGAUGAAAGAAUUACCGAAU
	AUUCGGUAAUUCUUUCAUCAC
siHIF-1α#2	CCUAUAUCCCAAUGGAUGAUG
	CAUCAUCCAUUGGGAUAUAGG
siHIF-2α#1	GCGCAAAUGUACCCAAUGAUA
	UAUCAUUGGGUACAUUUGCGC
siHIF-2α#2	CAGUACCCAGACGGAUUUCAA
	UUGAAAUCCGUCUGGGUACUG

Table S2. Vectors of plasmids.

cDNA	Vector	Source
NONO	pCDNA 3.1 TO Flag/HA	
SFPQ	pCDNA 3.1 HA	MiaoLing Plasmid Sharing Platform, Wuhan, China
PSPC1	pCDNA 3.1 TO HA	
HIF-1α	pCDNA 3.0 HA	Sino Biological, Beijing, China
HIF-2α	pCDH Flag	MiaoLing Plasmid Sharing Platform, Wuhan, China

Table S3. Primers for RIP

Gene	Primers (5'-3')
GLUT1	F: GTGTTCGGCCTGGACTCCATC
(exon)	R: CACTCTTGGCCCGGTTCTCCT
HK2	F: ACATAAGACCGTGCGGCGGCT
(exon)	R: CTGTGCCGTCCGGGGTAGCAC
LDHA	F: ATTCCTTTTGGTTCCAAGTCC
(exon)	R: CTTCATTAAGATACTGATGGCACA
ENO1	F: GTCTCTTCAGAGCTGCTGTGC
(exon)	R: CCTTCCCCATATAGCGAGTC
PGK1	F: GTTCCGCATTCTGCAAGC
(exon)	R: CTCATAACGACCCGCTTCC
VEGFA	F: TGGTGAAGTTCATGGATGTCTATCA
(exon)	R: CATGGTGATGTTGGACTCCTCA
VEGFB	F: CGAAGGTGACACATGGCTTTTCA
(exon)	R: CACAGTTCTTGTACCAAAGCCCAA
GLUT1	F: GACCAGTACCCGGAAACAAGG
(intron)	R: TGACCTCAAGCATTAAGTGCTCC
HK2	F: CACTACCAGGGAAGGCTCAGA
(intron)	R: CCAAGAAAAGTCACTGGAATTGG
LDHA	F: TCTTGATGCTGGTCAATATCACCT
(intron)	R: TGCAGATGTGGAAGCAGAACA
ENO1	F: ATATATTAGTTTAAAATGGAATGCTGA
(intron)	R: TGTCACGATCTCAGCTCACTG
PGK1	F: CCAGGCAGGAAGATTGCTTGA
(intron)	R: TTAGAAACGGTCTTGCTCTGTCAC
VEGFA	F: AAGCCTCTCTGCCTCAGGCGTT
(intron)	R: CCACTTGACATTTTAAGAGCTGATGGGT
VEGFB	F: GTGCCAGCCAGGCCCAACTTC
(intron)	R: TTGGTTCTACTCTTAGGAACCTGGTCCCCAG
snRNA U6	F: GTGCTCGCTTCGGCAGCACATA
	R: GAACGCTTCACGAATTTGCGTGTC

Table S4. Primers for Cut&Run

Gene	Primers (5’-3’)
GLUT1	GGGCTGTGTTACTCACTCTTACTCC
	CTCTTCCTGGGTTGTGTTCAAGCTG
LDHA	TTGGAGGGCAGCACCTTACTTAGA
	GCCTTAAGTGGAACAGCTATGCTGAC
ENO1	AAAGGCCCTGGACTCCCTTA
	AGGTGAACGTAAAGCCGGCGA
PDK1	CGCGTTTGGATTCCGTG
	CCAGTTATAATCTGCCTTCCCTATTATC
PKM2	TTCCTGCCTCTTGGTATGAC
	CGGCTTGTTCCCTCCTAC
VEGF	AGACTCCACAGTGCATACGTG
	AGTGTGTCCCTCTGACAATG
LOXL2	CACACATACACGTGCACACA
	AGGCTCTCCCCAAGGAAAT

Table S5. Antibody list

Antibody	Source	Catalog Number	Application in our study
NONO	Bethyl Laboratories	A300-587A	WB, PLA, Cut&Run, RIP, RIP-Seq
	Santa Cruz Biotechnology	sc-376865	PLA
HIF-1α	Proteintech	20960-1-AP	WB, PLA
	Abcam	ab1	PLA, Cut&Run
HIF-2α	Santa Cruz Biotechnology	sc-13596	PLA
	Abcam	Ab243861	WB, IHC
HIF-1β	Abcam	ab239366	WB, PLA
p300	Cell Signaling Technology	86377s	WB, PLA
CBP	Cell Signaling Technology	7389s	WB, PLA
SFPQ	Abcam	ab177149	WB, PLA
PSPC1	Santa Cruz Biotechnology	sc-374181	WB, PLA
HA-tag	Biolegend	901503	WB
Flag-tag	Proteintech	20543-1-AP	WB, COIP
VEGFA	Abcam	Ab1316	IHC
GLUT1	Abcam	ab115730	IHC
CD31	Biossci	PA1029	IHC
Ki67	Biossci	PA1007	IHC
Anti-Rabbit	Proteintech	SA00001-2	WB
	Abbkine	A25022	WB (for COIP sample)
Anti-Mouse	Proteintech	SA00001-1	WB

Table S6. Primers for RT-qPCR

Gene	Primer (5'-3')
Actin	F: CATGTACGTTGCTATCCAGGC
	R: CTCCTTAATGTCACGCACGAT
NONO	F: CTAGCGGAGATTGCCAAAGTG
	R: GTTCGTTGGACACATACTGAGG
HIF-1α	F: TGCTTGCCAAAAGAGGTGGA
	R: TTCTGTGTCGTTGCTGCCAA
GLUT1	F: ATGAACTACCCTCACTCCAGC
	R: TATTGGACACAGCTTGGATGCC
HK2	F: GAATGGGAAGTGGGGTGGAG
	R: GAGGAGGATGCTCTCGTCCA
LDHA	F: AGGAGAAACACGCCTTGATTTAG
	R: ACGAGCAGAGTCCAGATTACAA
ENO1	F: AAAGCTGGTGCCGTTGAGAA
	R: GGTTGTGGTAAACCTCTGCTC
CA9	F: TGAGGAAGGCTCAGAGACTCA
	R: GAGGCCAAAAACCAGGGCTA
BNIP3	F: AACTCAGATTGGATATGGGATTGG
	R: AGAGCAGCAGAGATGGAAGG
LOXL2	F: GGAAAGCGTACAAGCCAGAG
	R: GCACTGGATCTCGTTGAGGT
L1CAM	F: GCCACCTGTCATCACGGAAC
	R: GTCCAGCGGAACTGCACTTC
VEGFA	F: AGGGCAGAATCATCACGAAGT
	R: AGGGTCTCGATTGGATGGCA
